# Health related quality of life in Dutch young adults: psychometric properties of the PedsQL generic core scales young adult version

**DOI:** 10.1186/1477-7525-12-9

**Published:** 2014-01-18

**Authors:** Perrine F Limperg, Lotte Haverman, Hedy A van Oers, Marion AJ van Rossum, Heleen Maurice-Stam, Martha A Grootenhuis

**Affiliations:** 1 Psychosocial Department, Emma Children’s Hospital, Academic Medical Center, A3-241, Postbox 22660 1100 DD Amsterdam, the Netherlands; 2 Department of Pediatric Hematology Immunology & Infectious Diseases, Emma Children’s Hospital, Academic Medical Center, Postbox 22660, 1100 DD Amsterdam, the Netherlands; 3 Department of Rheumatology Reade Location Jan van Breemen 1056 AB Amsterdam, the Netherlands

**Keywords:** PedsQL, Young adults, Health related quality of life, Pediatrics, Psychometric properties

## Abstract

**Background:**

The purpose of this study is to provide Dutch norm data and to assess internal consistency and construct validity for the Pediatric Quality of Life Inventory Young Adult Generic Core Scales (PedsQL_YA) in Dutch young adults aged 18–30 years.

**Methods:**

A sample of 649 young adults from the general Dutch population aged 18–30 years, stratified by age, sex, marital status and education, completed a socio-demographic questionnaire and the Dutch version of the PedsQL_YA online. Internal consistency of the PedsQL_YA scales was determined with Cronbach’s alphas. Norm scores were obtained by calculating the mean PedsQL scale scores by gender, age and health status. Differences in scale scores were analyzed for gender, age and health status (construct validity) using two-sample t-tests and effect sizes were calculated. Construct validity was determined by testing differences in PedsQL scores between healthy young adults and young adults with chronic health conditions.

**Results:**

All scales of the PedsQL_YA showed satisfactory to excellent internal consistency, with Cronbach’s alphas between .77 and .94. Men reported higher scores (indicating better HRQOL) than women on all scales (p < .01), except for school/work functioning. No age differences were found. Young adults with chronic health conditions scored lower on all scales (p < .001) than healthy young adults, indicating good construct validity. Effect sizes varied from medium to large.

**Conclusions:**

The Dutch version of the PedsQL_YA has adequate psychometric properties. With the availability of reliable norm data, the PedsQL_YA can be used as a tool in the evaluation of health related quality of life in healthy young adults and those with a chronic health condition.

## Background

Health Related Quality of Life (HRQOL) as an outcome measure has received increased attention in pediatrics over the past years [1-4]. HRQOL is a multidimensional concept that refers to the impact of health and illness on an individual’s quality of life (QOL), which encompasses not only physical aspects but also social and emotional aspects [5]. The importance of these aspects has led to the development of instruments designed to measure HRQOL [1,6,7]. In pediatrics, the use of a generic HRQOL instrument provides the possibility to compare children across different chronic health conditions and to compare them to the healthy population [8-10]. So far, several age-appropriate measures are available to measure HRQOL for children up to 18 years old. Examples are the TNO AZL Preschool Children Quality of Life (TAPQOL) [11], the TNO AZL Children’s Quality of Life (TACQOL) [12], the Kidscreen [13], the Child Health Questionnaire (CHQ) [14] and the Pediatric Quality of Life Inventory (PedsQL) [15]. The PedsQL is considered one of the most frequently used generic HRQOL instruments for children and adolescents, and has the option of adding disease-specific modules to the generic core scales [16].

Research on children with chronic health conditions has shown that these children are at risk for HRQOL problems [17]. Due to advances in medicine, more children with chronic health conditions are now able to grow up as adults, which can lead to subsistent HRQOL problems in adulthood. Research on young adults (YAs) with chronic health conditions has shown that these YAs report lower HRQOL and achieve fewer milestones in independence, psychosexual and social development [3,18]. Moreover, it has been found that HRQOL of adult patients with chronic health conditions is frequently impaired [19]. Therefore, it is important to monitor HRQOL from childhood into adulthood. Routine HRQOL assessment can facilitate detection and discussion of psychosocial issues related to chronic health conditions. Moreover, it can provide a chance to refer patients with significant risks of an unfavorable psychosocial outcome for interventions and to help these patients to achieve optimal development [20,21].

To be able to monitor HRQOL over time in children moving from adolescence into adulthood, appropriate instruments and normative data are needed [6,22]. It is strongly recommended by the literature to use the same mode of administration when comparing groups or changes over time [23]. In order to provide continuity of HRQOL measurement with the same instrument from childhood into young adulthood, Varni & Limbers [6] adapted the items of the PedsQL 4.0 Generic Core Scales Adolescent Version for the use among young adults aged 18–25 years. This resulted in the PedsQL Young Adult Version (PedsQL_YA). The PedsQL_YA as a self-report measure of generic HRQOL has been found to have good feasibility, reliability and validity in an American university student population and discriminates between young adults with chronic health conditions and healthy young adults [6].

In the Netherlands, no HRQOL instruments that can be used from childhood into adulthood are available; norm data for the PedsQL 4.0 Generic Core Scales have only been collected for children from 5 until 18 years old [24]. Although the PedsQL_YA has been translated into Dutch by MAPI Institute (http://www.mapi-trust.org), psychometric properties of the Dutch PedsQL_YA have not been studied and norm data for young adults in the Netherlands are not available yet. With the increase of research on young adults with a chronic health condition and for use in clinical practice, norm data for this age group have become indispensable. To fill this gap, the aim of this study is to assess reliability and construct validity and provide Dutch norm data for the Dutch PedsQL_YA ages 18–30 years. With the approval of the authors of the PedsQL_YA [6], we broadened the age range from 18–25 years to 18–30 years, because the transition into adulthood is not always fulfilled at the age of 25 [25].

In line with former studies on the PedsQL_YA, we expect internal consistencies to be satisfactory. Regarding construct validity, we expect the PedsQL_YA to distinguish between healthy young adults and those with a chronic health condition. Age and gender differences are examined explorative.

## Methods

### Participants and procedures

Data collection of the PedsQL_YA was part of a large Dutch study aiming at establishing normative data for diverse questionnaires measuring various psychosocial concepts (Course of Life Questionnaire and PedsQL_YA Fatigue). Young adults were invited to participate in July 2012 and online data collection was carried out in cooperation with the Taylor Nelson Sofres Netherlands Institute for Public Opinion (TNS NIPO), a Dutch market research agency. TNS NIPO provides access to respondents of TNS NIPObase. TNS NIPObase is a database with a panel of 150,000 respondents who have indicated that they are willing to participate in TNS NIPO research on a regular basis. With the objective of obtaining at least 700 respondents, a stratified sample of 969 young adults in the age of 18–30 years was drawn from the panel. In order to meet the participation criteria for this study, the young adults had to be fluent in Dutch. TNS NIPO uses the software program ‘DIANA’ (http://www.niposoftware.com) for sampling and weighing procedures. The sample was stratified based on Dutch population figures regarding key demographics (age, sex, marital status and education). A stratified random sampling technique was used to minimize sample variance and to increase precision.

Prior to the data collection, informed consent was obtained from all participants. Responders were able to answer the questions online, on their own computer at home. Participants were told that the study was anonymous. The security of the website was guaranteed. The study was approved by the Medical Ethics Committee of the Academic Medical Centre, the Netherlands.

### Measures

#### Socio-demographics

To assess the socio-demographics of the participants, questions from the Course of Life Questionnaire (CoLQ) [26] were used regarding age, gender, ethnicity, education, employment and marital status. Education was divided into three categories according to the classification of Statistics Netherlands; low (primary education, lower vocational education, lower and middle general secondary education), intermediate (middle vocational education, higher secondary education, pre-university education), high (higher vocational education, university).

In addition, the respondents were asked about the presence and type of chronic health condition. The answers to this question were checked according to the definition by Mokkink et al. [27]. ‘The term ‘chronic health condition’ refers to conditions that can be determined with the help of medical-scientific knowledge and by means of valid measures, which cannot be cured (yet) and conditions that exist at least three months or have been present in three episodes for the past year’.

### PedsQL 4.0 (Pediatric Quality of Life Inventory) generic core scales young adult version

The PedsQL_YA is a generic self-report HRQOL instrument developed for young adults aged 18–25 years and contains 23 items in four scales; physical health (8 items), emotional functioning (5 items), social functioning (5 items) and work/school functioning (5 items). A psychosocial health scale score and a total scale score can be computed. The psychosocial health scale score (15 items) was computed by adding up the items of the emotional, social and work/school functioning scales and dividing them by the number of items answered. The total scale score was computed as the sum of all items divided by the number of items answered on all scales. Each of the 23 items states a problem, for example ‘it is hard for me to run’ and refers to the past week (acute version). On a 5-point Likert scale, the young adult indicates whether he/she had problems with that item. The Likert scale consists of the options ‘never’ (0), ‘almost never’ (1), ‘sometimes’ (2), ‘often’ (3) and ‘almost always’ (4). Each answer is reversed scored and rescaled to a 0–100 scale (0 = 100, 1 = 75, 2 = 50, 3 = 25 and 4 = 0). Higher scores on the PedsQL_YA indicate better reported HRQOL. Completing the PedsQL_YA takes approximately 5 minutes [6].

For this study, we used the PedsQL_YA provided by MAPI. This version was translated into Dutch according to the guidelines set by MAPI Research Trust, but not linguistically validated. After comparing this translated version to the validated Dutch version of the PedsQL for adolescents aged 13–18 years, several small differences were found: in the PedsQL_YA scale ‘social functioning’ the word ‘teenagers’ or ‘peers’ was substituted by ‘young adults’. Moreover, the scale ‘school functioning’ has been changed into ‘work/school functioning’. In this scale, the words ‘school’ or ‘class’ have been supplemented with ‘work’. The word ‘homework’ has been supplemented by ‘work or studies’. Because there were no linguistic differences between the Dutch MAPI PedsQL_YA and the Dutch PedsQL 13–18, we assume that the PedsQL_YA is linguistically valid. When comparing the Dutch PedsQL_YA to the English PedsQL_YA version, no differences in content were found.

### Statistical analyses

The Statistical Package for Social Sciences (SPSS) version 20.0 [28] for Windows was used for all statistical analyses. First, descriptive analyses were performed to describe the sample. To compare demographics of this sample to the stratified sample, we performed one sample t-tests (age) and binomial tests (gender and education as dichotomous variable). Furthermore, PedsQL scores were computed according to the PedsQL manual. The PedsQL data were normally distributed, so parametric tests were performed.

Second, to determine internal consistency of all PedsQL scales, Cronbach’s alpha coefficients were calculated based on average inter item correlation [29]. Cronbach’s alpha of < .70 was considered insufficient, ≥.70 was regarded as satisfactory and ≥ .80 as good. Scales with reliabilities of .70 or greater are recommended for comparing patient groups, while Cronbach’s alpha’s of .90 are recommended for analyzing individual patient scale scores [30].

Third, to provide norm scores, the mean PedsQL_YA scale scores with standard deviations were calculated by gender, age and health status (construct validity). In order to provide precise norm data, we split the sample into two age groups: 18–25 years and 26–30 years. We chose this division, based on the age group studied by Varni et al. [6]. Differences in PedsQL scores between the age groups and between men and women were analyzed using two-sample t-tests. To get insight into the extent of these differences, pooled effect sizes were calculated by dividing the difference in mean scores (18–25 versus 26–30 and women versus men) by the pooled standard deviation.

Finally, construct validity was determined by testing differences in the PedsQL scores between the healthy sample and young adults with a chronic health condition also using two-sample t-tests. In this case, effect sizes were calculated by dividing the difference in mean scores of healthy young adults and young adults with a chronic health condition by the standard deviation of the healthy sample.

All effect sizes of about 0.2 were considered small, effect sizes of about 0.5 medium and effect sizes of about 0.8 large [31].

## Results

### Socio-demographics

In total, 649 young adults (response rate 67%) participated. The key demographics (age, sex, marital status and education) of the respondents are comparable to the key demographics of the total stratified sample drawn by TNS NIPO. Table 1 represents the socio-demographics of the sample. The average age of the 332 women (51.2%) and of the 317 men (48.8%) was 24.79 years (SD 3.76).

**Table 1:** Socio-demographic characteristics

	**All participants**			**Healthy**			**Chronic**		
							**Health condition**^**1**^		
	**N**	**M**	**SD**	**N**	**M**	**SD**	**N**	**M**	**SD**
Age	649	24.79	3.76	512	24.70	3.70	137	25.14	3.98
	N	%		N	%		N	%	
Gender (female)	332	51.2		245	47.9		87	63.5	
Ethnicity (Dutch)	631	97.2		498	97.3		133	97.1	
Education^†^									
High	195	30.0		159	31.1		36	26.3	
Middle	322	49.6		254	49.7		68	49.6	
Low	131	20.2		98	19.2		33	24.1	
Employment (paid job)	448	69.0		361	70.5		87	63.5	
Marital status (married/living together)	236	36.4		183	35.7		53	38.7	

†Highest level completed: Low: primary education, lower vocational education, lower and middle general secondary education; Intermediate: middle vocational education, higher secondary education, pre-university education; High: higher vocational education, university.

^1^Most common conditions in Dutch sample were: asthma (34.3%), psychiatry (10.9%), gastro enterology (10.2%) and skin disease (5.8%).

Table 1 also shows socio-demographics of the sample split into young adults with a chronic health condition and the healthy sample. The sample included 512 (78.9%) Dutch healthy young adults (mean age 24.7 years, SD 3.70) and 137 young adults (21.1%) who indicated to have a chronic health condition (mean age 25.1 years, SD 3.98). No differences were found in key demographics, except for gender.

### Internal consistency

Table 2 presents the Cronbach’s alpha coefficients of the PedsQL scales for the total sample and for the sample split by gender, age and health status. In the total sample, Chronbach’s alphas ranged from .82 (social functioning) to .93 (total score). The Cronbach’s alphas of the PedsQL scales for the sample split by gender, age groups and health status ranged from .77 to .94.

**Table 2:** Internal consistency (Cronbach’s alpha) of the PedsQL (sub) scales

		**Total**		**Healthy**		**Chronic**	
						**Health condition**	
	**PedsQL (sub) scale**	**N**	**α**	**N**	**α**	**N**	**α**
**All participants**	Total score	649	.93	512	.92	137	.93
	Physical health	649	.89	512	.86	137	.91
	Psychosocial health	649	.91	512	.90	137	.90
	Emotional functioning	649	.84	512	.84	137	.83
	Social functioning	649	.82	512	.83	137	.79
	School/work functioning	649	.83	512	.81	137	.84
**Female**	Total score	332	.92	245	.90	87	.94
	Physical health	332	.90	245	.84	87	.92
	Psychosocial health	332	.90	245	.88	87	.91
	Emotional functioning	332	.83	245	.83	87	.83
	Social functioning	332	.82	245	.83	87	.80
	School/work functioning	332	.84	245	.80	87	.86
**Male**	Total score	317	.93	267	.93	50	.91
	Physical health	317	.87	267	.88	50	.82
	Psychosocial health	317	.91	267	.92	50	.88
	Emotional functioning	317	.84	267	.85	50	.81
	Social functioning	317	.82	267	.83	50	.77
	School/work functioning	317	.81	267	.81	50	.80
**Age group 18-25**	Total score	385	.92	310	.91	75	.92
	Physical health	385	.88	310	.85	75	.89
	Psychosocial health	385	.90	310	.90	75	.89
	Emotional functioning	385	.84	310	.84	75	.79
	Social functioning	385	.83	310	.83	75	.78
	School/work functioning	385	.82	310	.80	75	.79
**Age group 26-30**	Total score	264	.93	202	.93	62	.94
	Physical health	264	.91	202	.87	62	.93
	Psychosocial health	264	.91	202	.91	62	.92
	Emotional functioning	264	.85	202	.84	62	.86
	Social functioning	264	.81	202	.82	62	.80
	School/work functioning	264	.84	202	.82	62	.87

α Cronbach’s coefficient alpha.

### PedsQL scores

Table 3 contains the PedsQL scale scores of the total sample and by gender, age and health status. The mean total PedsQL score was 83.93 (SD 13.07) in the total sample.

**Table 3:** PedsQL norms: mean scale scores by gender, age and health status

		**Total**			**Healthy**			**Chronic**			**Chronic vs. healthy**	
								**Health condition**				
	**PedsQL (sub) scale**	**N**	**M**	**SD**	**N**	**M**	**SD**	**N**	**M**	**SD**	***p *** **value**	**Effect size**
**All participants**	Total score	649	83.93 ˜ ˜	13.07	512	85.88	11.45	137	76.65	15.92	.00***	0.81
	Physical health	649	87.13 ˜ ˜	16.01	512	89.60	12.99	137	77.90	21.87	.00***	0.90
	Psychosocial health	649	82.22 ˜ ˜	13.75	512	83.89	12.78	137	75.99	15.40	.00***	0.62
	Emotional functioning	649	77.23 ˜ ˜	18.04	512	78.54	17.60	137	72.30	18.87	.00***	0.35
	Social functioning	649	87.17 ˜	14.51	512	88.78	13.30	137	81.17	17.11	.00***	0.57
	School/work functioning	649	82.27	15.73	512	84.36	14.40	137	74.49	17.96	.00***	0.69
**Female**	Total score	332	81.27	13.30	245	84.10	10.70	87	73.30	16.40	.00***	1.01
	Physical health	332	83.61	17.61	245	87.53	12.89	87	72.59	23.57	.00***	1.16
	Psychosocial health	332	80.02	13.49	245	82.27	11.93	87	73.68	15.53	.00***	0.72
	Emotional functioning	332	72.86	17.56	245	74.39	17.05	87	68.56	18.33	.00***	0.34
	Social functioning	332	85.78	14.76	245	88.04	13.31	87	79.43	16.77	.00***	0.65
	School/work functioning	332	81.42	16.11	245	84.39	13.97	87	73.05	18.70	.00***	0.81
**Male**	Total score	317	86.72	12.23	267	87.51	11.88	50	82.48	13.30	.02*	0.42
	Physical health	317	90.81	13.20	267	91.50	12.82	50	87.13	14.69	.05	0.34
	Psychosocial health	317	84.53	13.66	267	85.38	13.37	50	80.00	14.44	.02*	0.40
	Emotional functioning	317	81.80	17.42	267	82.36	17.25	50	78.80	18.17	.21	0.21
	Social functioning	317	88.63	14.12	267	89.46	13.28	50	84.20	17.45	.02*	0.40
	School/work functioning	317	83.17	15.29	267	84.33	14.81	50	77.00	16.48	.01**	0.49
**Age group 18-25**	Total score	385	83.82°°	12.74	310	85.90	11.18	75	75.23	15.08	.00***	0.95
	Physical health	385	87.78°°	15.16	310	90.20	12.45	75	77.79	20.47	.00***	1.00
	Psychosocial health	385	81.71°	13.66	310	83.60	12.68	75	73.78	14.83	.00***	0.77
	Emotional functioning	385	76.73°°	18.07	310	78.42	17.68	75	69.73	18.08	.00***	0.49
	Social functioning	385	86.64	14.90	310	88.44	13.66	75	79.20	17.42	.00***	0.68
	School/work functioning	385	81.75	15.45	310	83.95	14.26	75	72.67	16.89	.00***	0.79
**Age group 18–25 female**	Total score	206	81.95	12.94	159	84.73	10.74	47	72.55	15.24	.00***	1.13
	Physical health	206	85.48	16.09	159	88.95	12.05	47	73.74	21.73	.00***	1.26
	Psychosocial health	206	80.06	13.59	159	82.47	12.23	47	71.91	14.57	.00***	0.86
	Emotional functioning	206	72.52	18.05	159	74.62	17.86	47	65.43	17.28	.00***	0.51
	Social functioning	206	85.85	14.99	159	88.11	13.58	47	78.19	17.05	.00***	0.73
	School/work functioning	206	81.82	15.49	159	84.69	13.99	47	72.13	16.51	.00***	0.90
**Age group 18–25 male**	Total score	179	85.97	12.20	151	87.13	11.54	28	79.74	13.92	.01**	0.64
	Physical health	179	90.43	13.58	151	91.51	12.77	28	84.60	16.36	.04*	0.54
	Psychosocial health	179	83.59	13.54	151	84.79	13.07	28	77.14	14.44	.01**	0.59
	Emotional functioning	179	81.56	16.89	151	82.42	16.72	28	76.96	17.34	.13	0.33
	Social functioning	179	87.54	14.78	151	88.77	13.77	28	80.89	18.21	.04*	0.57
	School/work functioning	179	81.68	15.44	151	83.18	14.54	28	73.57	17.79	.01**	0.66
**Age group 26-30**	Total score	264	84.09^^^	13.55	202	85.85	11.87	62	78.37	16.85	.00***	0.63
	Physical health	264	86.17^^^	17.17	202	88.68	13.76	62	78.02	26.63	.00***	0.77
	Psychosocial health	264	82.98^^^	13.86	202	84.34	12.96	62	78.55	15.79	.00***	0.45
	Emotional functioning	264	77.95^^^	18.00	202	78.74	17.51	62	75.40	19.47	.20	0.19
	Social functioning	264	87.95^^	13.92	202	89.31	12.75	62	83.55	16.56	.01**	0.45
	School/work functioning	264	83.03^	16.13	202	84.98	14.62	62	76.69	19.08	.00***	0.57
**Age group 26–30 female**	Total score	126	73.41	16.78	86	82.94	10.59	40	74.18	17.83	.00***	0.83
	Physical health	126	85.67	14.11	86	84.88	14.02	40	71.25	25.78	.00***	0.97
	Psychosocial health	126	80.75	17.12	86	81.90	11.41	40	75.75	16.22	.02*	0.54
	Emotional functioning	126	79.95	13.37	86	73.95	15.70	40	72.25	19.05	.60	0.11
	Social functioning	126	80.16	13.86	86	87.91	12.87	40	80.88	16.52	.01**	0.55
	School/work functioning	126	80.56	19.53	86	83.84	13.99	40	74.13	21.15	.00***	0.69
**Age group 26–30 male**	Total score	138	82.10	18.14	116	88.01	12.34	22	85.97	11.85	.48	0.17
	Physical health	138	90.04	13.13	116	91.49	12.93	22	90.34	11.85	.70	0.09
	Psychosocial health	138	85.11	17.93	116	86.15	13.77	22	83.64	13.92	.44	0.18
	Emotional functioning	138	85.75	13.77	116	82.28	17.99	22	81.14	19.33	.78	0.06
	Social functioning	138	87.68	12.25	116	90.34	12.61	22	88.41	15.84	.53	0.15
	School/work functioning	138	91.30	12.73	116	85.82	15.08	22	81.36	13.82	.20	0.30

*p < .05, **p < .01, ***p < .001 chronic versus healthy.

˜p < .01, males differed from females in the total group.

˜ ˜p < .00, males differed from females in the total group.

°p < .01, males differed from females in age group 18–25.

°°p < .00, males differed from females in age group 18–25.

^p < .03, males differed from females in age group 26–30.

^^p < .01, males differed from females in age group 26–30.

^^^p < .00, males differed from females in age group 26–30.

#### Gender

The mean of the total PedsQL score by gender (not split by age and health status), was 81.27 (SD 13.30) for women and 86.72 (SD 12.23) for men. Men reported higher scores than women on all scales (p < .01, effect sizes .20 to .51), except for school/work functioning*.*

#### Age

The mean of the total PedsQL score by age (not split by gender and health status) was 83.82 (SD 12.74) for age group 18–25 years and 84.09 (SD 13.55) for 26–30 years. When comparing the two age groups, no differences were found in any of the PedsQL scale scores.

#### Age * gender

When looking at age group 18–25 years, men scored significantly higher on all scales than women (p < .01, effect sizes .26 to .52), except for social and school/work functioning. In the age group 26–30 years, men scored higher on all scales than women (p < .03, effect sizes .27 to .66).

### Construct validity

#### Health status

The mean of the total PedsQL score for the healthy sample was significantly higher on all scales (p < .001, effect sizes .35 to .90), than the group of young adults with a chronic health condition. The mean of the total PedsQL score for the healthy sample was 85.88 (SD 11.45) and 76.65 (SD 15.92) for the chronic health condition.

#### Health status * gender

When looking at health status by gender, the healthy women scored significantly higher on all scales than women with a chronic health condition (p < .00, effect sizes .34 to 1.16). The healthy men scored significantly higher on four (total score, physical health, social and school/work functioning) out of six scales than men with a chronic health condition (p < .02, effect sizes .40 to .49). No differences were found on the scales physical health and emotional functioning.

#### Health status * age

When looking at health status by age, the healthy young adults aged 18–25 years scored significantly higher on all scales than the young adults aged 18–25 years with a chronic health condition (p < .00, effect sizes .49 to 1.00). The healthy young adults aged 26–30 years scored significantly higher on all scales than young adults aged 26–30 years with a chronic health condition (p < .01, effect sizes .45 to .77), except for emotional functioning.

#### Health status * age * gender

When looking at health status by gender in the age group 18–25 years, the healthy women and men reported better HRQOL on all scales than respectively women and men with a chronic health condition (p < .05, effect sizes .51 to 1.26,), except for emotional functioning in men. When looking at health status by gender in the other age group (26–30 years), the healthy women reported better HRQOL on all scales than women with a chronic health condition (p < .02, effect sizes .54 to .97), except for emotional functioning. In men aged 26–30 years, no differences in HRQOL scores were found between the healthy sample and the men with a chronic health condition.

## Discussion

Based on the data of 649 young adults aged 18–30 years old, we conclude that the Dutch version of the PedsQL_YA has adequate psychometric properties and can be used as an HRQOL measurement instrument for healthy YAs and YAs with a chronic health condition in the Netherlands.

The PedsQL_YA internal consistency reliabilities all exceed the recommended minimum alpha coefficients standard of .70 for group comparisons. Moreover, the total scale scores exceed an alpha of .90, recommended for analyzing individual patient scales [30,32]. The Dutch sample shows similar results in reliability across scales to the US sample [6], with slightly higher alphas. As far as we know, the PedsQL Generic Core scales is the only generic quality of life instrument to span ages 5–30 years in the Netherlands for self-report while maintaining scale construct consistency [6].

With respect to construct validity, the PedsQL_YA differentiates between healthy young adults and young adults with a chronic health condition, in line with our hypothesis. In accordance with previous studies [6,15], young adults with a chronic health condition, report lower functioning in all HRQOL domains than their healthy peers. The lower emotional and social functioning of YAs with chronic health conditions may reflect the reduced social participation and delayed achievement of psychosocial developmental milestones in these YAs, compared to their healthy peers [18,33]. The lower HRQOL regarding school/work functioning might be explained by the typically higher rates of absences for individuals with chronic health conditions compared to healthy populations [34]. It has been found that YAs with chronic health conditions are less able to work than their healthy peers and have paid jobs less often [35,36]. Surprisingly, the differences described above seem not to apply for men ages 26–30. This finding might be explained by the small number of respondents in the subgroup of young men ages 26–30 with a chronic health condition.

The present findings of young adult men reporting better HRQOL than young adult women are in line with previous literature [2,6,23]. The gender differences seem to reflect a true dissimilarity between men and women and therefore give further evidence for the validity of the PedsQL_YA as a sensitive measure of measuring HRQOL in YAs.

Unfortunately, limited research has been done on comparing HRQOL in young adults up to 25 years old and older adults. In our study no differences were found in HRQOL scores between the two age groups 18–25 and 26–30 years. Studies about young adults with a history of cancer suggest that young adults ages 18–25 years report better HRQOL than older young adults [37,38]. So, further research is needed to study the differences in HRQOL between these specific aged groups (18–25 and 26–30) from the general population.

A strong point of our study is that the PedsQL norm data collected in this study are an adequate representation of the general Dutch population due to the stratified sampling. Moreover, we have a large sample size without any missing data. The online method of data collection explains this, with missing values not being allowed. It has been shown that online data collection increases response rates and data quality [39].

Despite the strengths mentioned above, the present study has several limitations that need to be taken into account. First, the reliability of the assessment of the health condition status was based on self-report rather than on physician-report. However, according to literature, self-reports of health status are consistent with proxy-reports of patient health status, including physician diagnoses of chronic health conditions [40]. Second, it is possible that more severely ill young adults did not participate in the study because they were not able to sit behind the computer and complete questionnaires due to the severity of their health condition or did not want to burden themselves with participation. Therefore, it is possible that our sample contains a slightly different type of chronic condition than encountered in clinical practice. Our study shows that 21% of young adults have a chronic health condition, which is comparable to 22.4% in the general population [41]. Third, even though nearly 700 participants were included, sample sizes were relatively small for some subgroups.

Based on the results of our study, we conclude that YAs with chronic health conditions experience lower HRQOL than their healthy peers. Especially for those YAs transitioning from pediatrics to adult healthcare, the PedsQL_YA can be of great added value. For all children, transition into adulthood is a critical phase. In addition, we know that stress of transition from childhood to adulthood is heightened for those with chronic health conditions [42]. Moving from pediatric to adult healthcare is an essential process in the lives of all young people with chronic health conditions and can be a demanding life event, as they must move from parental control of their healthcare needs to self-care [43,44]. By using electronic Patient Reported Outcomes (ePROs), such as the feedback of HRQOL questionnaires, in daily clinical practice, doctors, psychologists and nurses can identify specific problems in HRQOL domains of YAs with chronic health conditions [45,46]. Therefore, we suggest that both the medical and psychosocial situation of YAs with a chronic health condition should be monitored systematically by their healthcare providers. Consequently, more tailored care, guidance and advice can be given to these YAs. An example of a system to systematically monitor HRQOL is KLIK (http://www.hetklikt.nu/englishdemo), which is a web-based system to monitor HRQOL in daily clinical practice for children and YAs with chronic health conditions in the Netherlands. The Dutch PedsQL_YA is a suitable instrument for systematic HRQOL assessment in daily clinical practice, since this questionnaire is short and easy to administer [6,46].

## Conclusions

In conclusion, the Dutch version of the PedsQL_YA Generic Core Scales demonstrates overall adequate psychometric properties. With the obtained norm data, the PedsQL_YA can be utilized as a tool to evaluate HRQOL in young adults.

## Abbreviations

HRQOL: Health related quality of life; QOL: Quality of life; TAPQOL: TNO AZL Preschool Children Quality of Life; TACQOL: TNO AZL Children’s Quality of Life; CHQ: Child Health Questionnaire; PedsQL: Pediatric Quality of Life Inventory; PedsQL_YA: Pediatric Quality of Life Inventory Young Adult Generic Core Scales; TNS NIPO: Taylor Nelson Sofres Netherlands Institute for Public Opinion

## Competing interests

The authors declare that they have no competing interests.

## Authors’ contributions

PL carried out the literature study, data collection, data analysis and drafted the manuscript. LH conceived the study, carried out data collection, data analysis and critically revised the manuscript. HvO carried out data collection and critically revised the manuscript. MvR critically revised the manuscript for intellectual content. HMS performed data analysis and critically revised the manuscript. MG supervised data collection and data analysis, and critically revised the manuscript. All authors read and approved the final manuscript.

## Authors’ information

PL is a psychologist and PhD student at the Pediatric Psychosocial Department of the Emma Children’s Hospital Academic Medical Center (AMC) Amsterdam.

LH is a psychologist and postdoctoral researcher at the Pediatric Psychosocial Department of the Emma Children’s Hospital Academic Medical Center (AMC) Amsterdam.

HvO is a psychologist and PhD student at the Pediatric Psychosocial Department of the Emma Children’s Hospital Academic Medical Center (AMC) Amsterdam.

MvR is a pediatric rheumatologist at the Department of Pediatric Hematology Immunology & Infectious Diseases of the Emma Children’s Hospital Academic Medical Center (AMC) as well as at the Department of Rheumatology Reade Location Jan van Breemen in Amsterdam.

HMS is health scientist and postdoctoral researcher within the Pediatric Psychosocial Department of the Emma Children’s Hospital Academic Medical Center (AMC) Amsterdam who provides methodological support.

MG is a professor in pediatric psychology and head research of the pediatric psychology program in the Emma Children’s Hospital which is directed at three principal areas: studying the effects of a chronic disease or life-threatening disease on quality of life of children and young adults and family members; finding factors which predict these outcomes and development, implementation and evaluation of intervention programs. The department has extensive research experience in coordinating randomized controlled trials of psychosocial cognitive behavioral interventions with children with chronic diseases and cancer, and developing web-based interventions for young cancer survivors and their parents.
